# Case Report: IgA Nephropathy in a Patient With Anti-Transcription Intermediary Factor-1γ Antibody-Positive Dermatomyositis

**DOI:** 10.3389/fimmu.2022.757802

**Published:** 2022-02-03

**Authors:** Suo Zhang, Yu-Lan Chen, Cui-Lian Liu, Jing-Yi Xie, Bao-Dong Sun, Dong-Zhou Liu

**Affiliations:** ^1^ Department of Rheumatology and Immunology, Shenzhen People’s Hospital (The Second Clinical Medical College, Jinan University, The First Affiliated Hospital, Southern University of Science and Technology), Shenzhen, China; ^2^ Department of Rheumatology and Immunology, Shenzhen Second People’s Hospital, Shenzhen, China

**Keywords:** IgA nephropathy, systemic autoimmune myopathies, dermatomyositis, anti- transcription intermediary factor-1γ antibody, dermatomyositis-specific antibodies

## Abstract

Immunoglobulin A nephropathy (IgAN) is the most common primary glomerulonephritis characterized by IgA deposits in the mesangial area of glomeruli. Connective tissue disorders are some of the most frequent causes of secondary IgAN. Nevertheless, IgAN rarely occurs in systemic autoimmune myopathies (SAMs). The present case study reports on a 58-year-old patient with dermatomyositis with positive anti-transcription intermediary factor (TIF)-1γ antibodies who was diagnosed with IgAN during standard immunosuppressive therapy. Moreover, we have made a systematic review regarding the association of SAMs and IgAN. To the best of the authors’ knowledge, this is the first case study describing a patient with anti-TIF1γ antibody-positive dermatomyositis who developed IgAN, which demonstrates a potential relationship between anti-TIF1γ-positive dermatomyositis and IgAN. It is important for clinicians to be aware of the possibility of renal involvement in patients with SAMs, even in those with anti-TIF1γ-positive dermatomyositis.

## Introduction

Systemic autoimmune myopathies (SAMs) are a complex heterogeneous group of diseases characterized by muscle inflammation and involve extramuscular organs, including the skin, joints, lungs, heart, and gastrointestinal tract (1, 2). Dermatomyositis (DM) and polymyositis (PM) are two main types of SAMs. Unlike respiratory and gastrointestinal involvement, renal involvement rarely occurs in SAMs compared with other autoimmune diseases, such as systemic lupus erythematosus and scleroderma (3). Immunoglobulin A nephropathy (IgAN) is the most common primary glomerulonephritis characterized by IgA deposits in the mesangial area of glomeruli. Connective tissue disorders are some of the most frequent causes of secondary IgAN (4). Nevertheless, SAM-associated IgAN has rarely been reported. Here, to the best of our knowledge, we describe for the first time a 58-year-old patient with DM with positive anti-transcription intermediary factor (TIF)-1γ antibodies who developed IgAN during standard immunosuppressive therapy.

## Case Presentation

A 58-year-old Chinese man was admitted to our hospital in June 2020 due to a six-year history of a recurrent rash and a new onset of haematuria and proteinuria. In June 2014, the patient was diagnosed with DM based on the presence of a heliotrope rash and Gottron’s papules, progressive proximal muscle weakness with a significant elevation in creatine kinase (CK 1867 U/L, reference range 25-192), and proximal myopathic changes indicated by electromyography (5). The routine urinalysis was normal. Methylprednisolone (80 mg/day), methotrexate (12.5 mg/week) and cyclophosphamide (400 mg/week) were initiated. However, the patient developed a pulmonary cytomegalovirus infection within a month (July 2014) and therefore was changed from methotrexate and cyclophosphamide to tacrolimus (4 mg/day). His DM was stable without any rash or muscle weakness for five years with low-dose prednisolone (5 mg/day) and tacrolimus (2 mg/day) for maintenance therapy.

In May 2019, the patient again developed a heliotrope rash and proximal muscle weakness, with a mild elevation in CK (362 U/L). His routine urinalysis was normal. Further tests revealed that his anti-TIF1γ antibodies were positive based on an immune blotting assay. A positron emission tomography-computer tomography (PET-CT) was unremarkable. There was no evidence to suggest infection or malignancy. Therefore, the methylprednisolone dosage was increased to 40 mg/day and the tacrolimus dosage was increased to 3 mg/day, resulting in gradual improvement of his rash. In April 2020, the methylprednisolone was gradually tapered until it was discontinued, and tacrolimus (3 mg/day) was continued for maintenance therapy. In June 2020, the patient’s rash reoccurred, and there was a new onset of haematuria (3+) and proteinuria (2+) on the urinalysis. He denied other past medical history, including diabetes mellitus, hypertension, and nephropathies.

Physical examination on admission revealed the presence of a heliotrope rash and Gottron’s papules (**Supplementary Figures 1A, C**) without any signs of muscle weakness or pain. Laboratory tests revealed the following metabolic levels: CK at 171 U/L, lactate dehydrogenase (LDH; reference range 110-240) at 281 U/L, alanine aminotransferase (ALT; reference range 0-40) at 85 U/L, aspartate transaminase (AST; reference range 0-40) at 59 U/L, C-reactive protein (CRP; reference range <5) at 3.46 mg/L, erythrocyte sedimentation rate (ESR; reference range 0-20) at 30 mm/h, ferritin (reference range 11.0-306.8) at 708.7 ng/mL, immunoglobulin A (reference range 0.7-4.5) at 6.25 g/L, serum urea nitrogen (BUN; reference range 2.5-7.5) at 3.99 mmol/L, and creatinine (Cr; reference range 44-133) at 58 µmol/L. A urinalysis revealed a proteinuria (0.3 g/L) with a strongly positive haematuria with a 24-hour urine protein (reference range 0.028-0.141) of 0.94 g. Further assessment of urinary red blood cell morphology by phase-contrast microscopy showed that the urinary red blood cell count was 35854/ml, with only 22% normal urinary red blood cells, and there was no evidence of malignancy, diabetes, hypertension or urolithiasis. The main laboratory results are shown in **Supplementary Table 1**.

**Figure 1:**
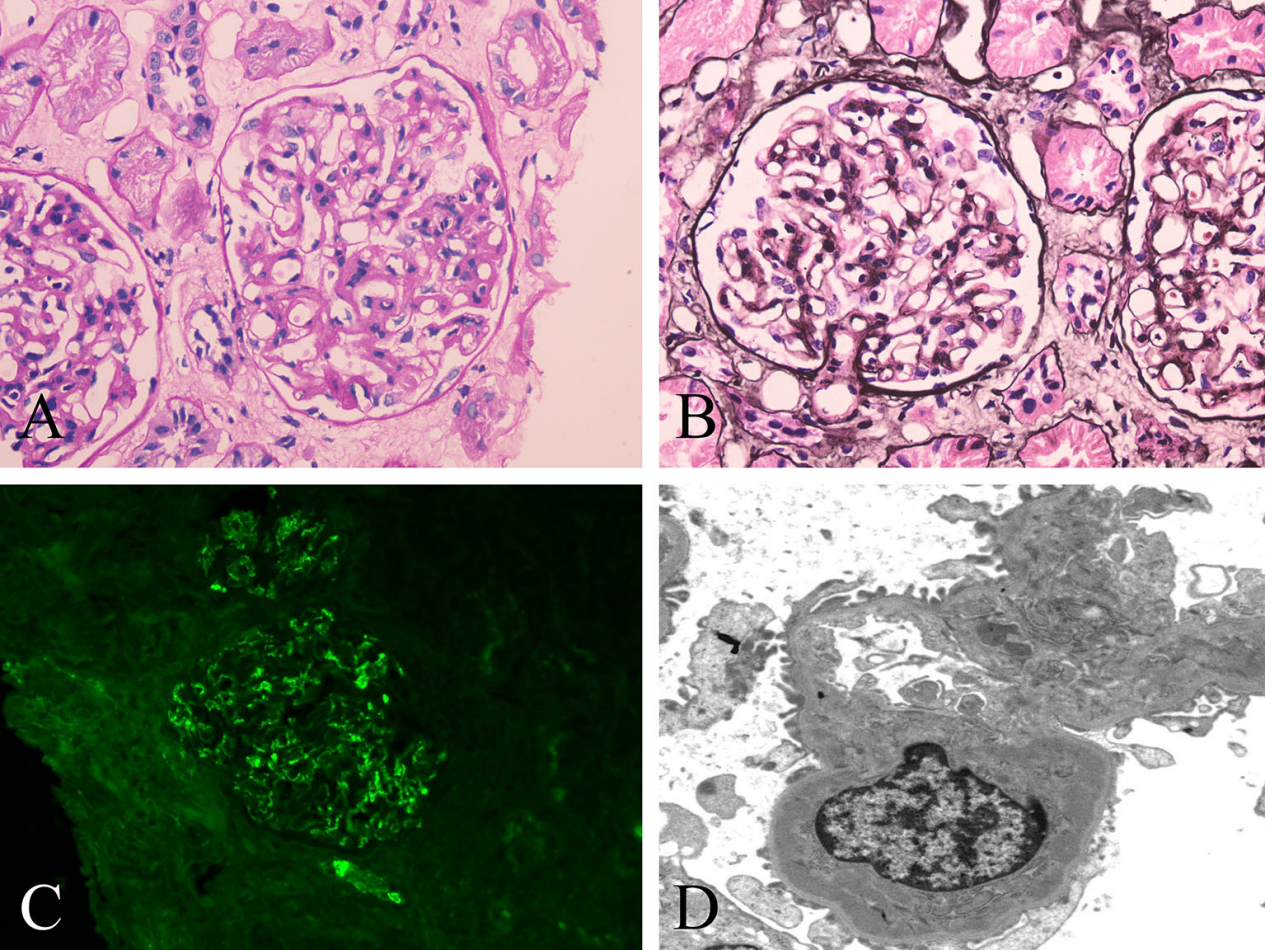
Histopathological changes of renal biopsy in the patient. **(A, B)** Light microscopy shows a glomerulus with mild to moderate mesangial hypercellularity and endocapillary hypercellularity. **(A)** Periodic acid-Schiff staining; original magnification ×400. **(B)** Periodic acid-silver methenamine staining; original magnification ×400. **(C)** IgA deposits along the glomerular mesangial area by immunofluorescence. original magnification ×400. **(D)** Deposits along the mesangium and matrix by electron microscopy. original magnification ×5000.

A percutaneous renal biopsy was performed to confirm nephropathy. The renal biopsy revealed that one was globally sclerotic among 30 glomeruli sampled. The remainder had mild to moderate mesangial hypercellularity, accompanied by excessive intracapillary cells (**Figures 1A, B**). Segmental sclerosis, interstitial fibrosis or tubular atrophy, or cellular or fibrocellular crescent could not be found in the renal biopsy. Immunofluorescence staining demonstrated proliferative mesangial deposits of IgA (3+) (**Figure 1C**), C3 (2+), IgM (1+), kappa (1+), and lambda (2+). In addition, electron-dense deposits in the mesangium and matrix were also detected by electron microscopy (**Figure 1D**). The Oxford MEST-C score (M: mesangial hypercellularity; E: endocapillary hypercellularity; S: segmental sclerosis; T: percentage of interstitial fibrosis or tubular atrophy; C: active cellular or fibrocellular crescent) was M1E1S0T0C0 according to the Oxford classification of IgAN (6). These findings led to the diagnosis of a DM flare complicated by IgAN. Therefore, methylprednisolone (40 mg/day) and methotrexate (10 mg/week) with valsartan were initiated for the patient, and tacrolimus was discontinued. During the follow-up, the patient’s rash gradually improved. After 3 months of immunosuppressive treatment, the patient’s rash substantially improved (**Supplementary Figures 1B, D**), with normal levels of CK, LDH, ALT, and AST. His 24-hour urine protein decreased to 0.640 g, but red blood cells were still found on the urinalysis. At the time of writing this report, the patient was being treated with 6 mg/day methylprednisolone, 15 mg/week methotrexate and valsartan, and his DM was stable with a minimal rash on his face and neck. The clinical course of the patient is shown in **Table 1**.

**Table 1:** Timeline of the disease and treatment.

Timeline	Symptoms/diagnosis	Treatment	Outcome
June 2014	Heliotrope rash, Gottron’s papules, muscle weakness with elevated creatine kinase, and myopathic changes indicated by electromyography; normal urinalysis	mPSL (80 mg/day), MTX (12.5 mg/week) and CTX (400 mg/week) as initial therapy	Improved
July 2014	Pulmonary cytomegalovirus infection; normal urinalysis	Changed MTX and CTX to tacrolimus (4 mg/day); low-dose prednisolone (5 mg/day) and tacrolimus (2 mg/day) for maintenance therapy	Improved and stable for 5 years
May 2019	Heliotrope rash and muscle weakness with elevated CK, positive anti-TIF1γ antibodies; normal urinalysis	mPSL (40 mg/day) and tacrolimus (3mg/day); mPSL was tapered until discontinued, and tacrolimus (3 mg/day) was remained for maintenance therapy	Improved
June 2020	Heliotrope rash and Gottron’s papules without muscle weakness; new-onset of hematuria and proteinuria, and biopsy-proven IgAN	Tacrolimus was stopped; mPSL (40 mg/day), MTX (10 mg/week) and valsartan as initial therapy; mPSL (gradually tapered to 6 mg/day), MTX (15 mg/week) and valsartan at the time of writing this report	Rash improved, 24-hour urine protein decreased, but urine red blood cells could still be found

IgAN, IgA nephropathy; anti-TIF1γ, anti-transcription intermediary factor; mPSL, methylprednisolone; MTX, methotrexate; CTX, cyclophosphamide.

## Discussion

SAMs consist of five main forms: DM, PM, anti-synthetase syndrome, immune-mediated necrotizing myopathy and inclusion body myositis (2). Over the last few decades, several DM-specific autoantibodies have been discovered, such as anti-Mi2, anti-nuclear matrix protein (NXP)2, anti-TIF1γ, anti-small ubiquitin-like modifier activating enzyme (SAE) and anti-melanoma differentiation-associated protein (MDA)5 antibodies (7). Approximately 70% of patients with DM have DM-specific autoantibodies, and each of these has been associated with a unique clinical phenotype (8). TIF1γ, encoded by the tripartite motif containing (TRIM) 33 gene, is involved in various biological pathways, such as transforming growth factor (TGF)-β signaling pathway and Wnt/β-catenin pathway (9). The relationship between anti-TIF1γ antibodies and cancer in patients with DM has been well established. As many as 50% of anti-TIF1γ-positive DM patients have developed cancers within 3 years from the onset of myositis (7). The pathogenic mechanism of the anti-TIF1γ antibody is not yet clear and may be related to the tumorigenic role of the TIF1 family proteins (10). In this study, a diagnosis of anti-TIF1γ antibody-positive DM was established in our patient. However, there was no evidence of malignancies in the patient during the six-year follow-up. Intriguingly, a new onset of IgAN was found in the patient. To the best of our knowledge, we describe for the first time that a DM patient with anti-TIF1γ antibodies developed IgAN during standard immunosuppressive therapy. Our case study highlights a potential association between anti-TIF1γ-positive DM and IgAN, suggesting the need for close monitoring of new-onset IgAN during the course of the preexisting DM for an early diagnosis and management of IgAN. Renal biopsy is advisable in patients who manifest with persistent renal abnormalities during the follow-up. It is important for clinicians to be aware of the possibility of renal involvement in patients with SAMs, even in those with anti-TIF1γ-positive DM rather than only considering the association with malignancy.

Renal involvement is rare in SAMs. The most common renal involvement includes acute tubular necrosis and chronic glomerulonephritis (11, 12). A multicentre study including 150 patients with SAMs (DM, PM, or anti-synthetase syndrome) showed that renal involvement occurred in 35 patients (23.3%): acute kidney injury in 16 patients (10.7%) and chronic kidney disease in 31 patients (20.7%). The main cause of acute kidney injury was acute tubular necrosis caused by drugs or myoglobinuria (11). Yen et al. (12) reported that 14 out of 65 patients with PM/DM had renal involvement. Takizawa et al. (13) reported 21 cases of PM/DM-associated glomerulonephritis confirmed by biopsy. The diagnosis included 12 cases of glomerular proliferative glomerulonephritis, six cases of membranous glomerulonephritis, one case of minimal change glomerulonephritis and two cases of crescentic glomerulonephritis. A common finding of PM is mesangial proliferative glomerulonephritis, while that of DM is membranous glomerulonephritis. However, IgAN is rarely reported in SAMs according to the previously published literature, which was confirmed in our patient based on the biopsy.

IgAN is the most common primary glomerulonephritis in the world. Renal histopathology has shown that IgA was the major immunoglobulin in glomeruli, usually accompanied by complement C3, and often accompanied by IgG and IgM (4). Abnormal glycosylation of the IgA1 molecule has been reported to be the key cause of IgAN. Abnormal IgA1 molecules polymerize or bind with IgG or IgA1 antibodies *in vivo* as autoantigens to form immune complexes, which promote inflammatory reactions and complement activation. Complement activation can also increase the direct damage to podocytes caused by various cytokines or chemokines. Notably, an *in vitro* study has revealed a decreased activity of β-1,3-galactosyltransferase that is essential in IgA1 glycosylation in IgA1-producing cells, which contributes to hypoglycosylation of IgA1 in patients with IgAN (14). Secondary IgAN is mainly reported in IgA vasculitis, liver diseases, chronic infections, and tumors (4, 15). In addition, IgAN may also be associated with a variety of rheumatic diseases, including inflammatory bowel disease (IBD) (16), spondyloarthritis (17), systemic lupus erythematosus (18), Sjögren syndrome (19) and rheumatoid arthritis (RA) (20). Case reports of PM/DM complicated with IgAN are not common. To date, 11 cases of SAMs with IgAN have been reported previously: two cases of juvenile DM (21, 22), two DM (23, 24), three PM (25–27), and four anti-synthetase syndrome (11, 13, 28). The details of the reported 11 cases are shown in **Table 2**. Current studies have revealed a similar galactose-deficient IgA1-mediated pathogenesis in secondary IgAN (15). Furthermore, the pathogenesis of IgAN in Sjögren syndrome is probably attributed to the deposition of IgA-containing circulating immune complexes that are secreted by activated monoclonal B lymphocytes and the dysregulation of polymeric IgA (19). However, the relationship between IgAN and DM is still unclear. It is more likely that the two immune diseases may have similar immune pathogenic mechanisms (21), both of which are mainly mediated by humoral immune system. Nevertheless, the relationship between IgAN and PM seems to be unclear, in which cellular immunity is considered to be the dominant mechanism (26).

**Table 2:** Case reports regarding systemic autoimmune myopathies with IgA nephropathy.

Ref	Sex/age	Myopathy	Concomitant disease	Time^#^	Antibody	Treatment	Outcome
(21)	F/14	JDM	None	0	Negative	GCs, MTX	Improved
(22)	M/10	JDM	None	0	Negative	GCs, MTX	Improved
(23)	F/26	DM	None	1.5 years	NA	GCs, AZA	NA
(25)	M/32	PM	Scleroderma, adult coeliac disease and diabetes mellitus	1 year	RF, anti-nucleolus, anti-reticulin	GCs	Improved
(24)	M/49	DM	Pulmonary fibrosis and lung cancer	0	Negative	GCs	Dead
(26)	M/35	PM	Acute lung injury	6 months	Negative	GCs, AZA, CYC	Improved
(27)	F/56	PM	Thyroid papillary carcinoma	0	ANA (speckled pattern, 1:320)	GCs, CYC, IVIG, total thyroidectomy	Improved
(13)	M/58	ASS	Interstitial lung disease	0	Anti-Jo-1	GCs, IVIg, CSA	Improved
(28)	F/65	ASS	Lung cancer	0	Anti-Jo-1	Surgical treatment of lung cancer	Improved
(11)	F/38	ASS	None	0	Anti-Jo-1, anti-SSA, anti-SSB,	GCs, MTX, IVIg, TNF-i	Improved
(11)	M/43	ASS	None	18 years	Anti-Jo-1, anti-SSA	GCs, MTX	Improved

Ref, reference; NA, not available; JDM, juvenile dermatomyositis; DM, dermatomyositis; PM, polymyositis; ASS, anti-synthetase syndrome; GCs, glucocorticosteroids; MTX, methotrexate; AZA, azathioprine; CYC, cyclophosphamide; CyA, cyclosporine A; MMF, mycophenolate mofetil; RF, rheumatoid factor; IVIg, intravenous immunoglobulin; TNF-i, TNF-α inhibitor; M, male; F, female.

^#^Time between myositis onset and IgA nephropathy.

Intriguingly, our patient developed IgAN during standard immunosuppressive therapy, which is the first case report describing the development of IgAN in a DM patient with anti-TIF1γ antibodies. However, IgAN is known as a “multi-hit hypothesis” of pathogenesis (29), and the mechanism by which TIF1γ may be associated with the development of IgAN is still unclear. TIF1γ, as a member of TRIM family, has E3 ubiquitinprotein ligase activity (9), which may be involved in the degradation of the specific glycosyltransferases in IgA1 glycosylation, such as β-1,3-galactosyltransferase mentioned above. Moreover, anti-TIF1-γ antibodies have been reported to be specially associated with human leucocyte antigen (HLA)-DQB1*02 in adult-onset patients (30). Genome-wide association analyses of IgAN identified HLA-DQB1 as one of the loci with high susceptibility (31, 32). Therefore, a similar genetic predisposition may partially explain the relationship between anti-TIF1γ antibody and IgAN. In addition, an active immune response could directly or indirectly contribute to inducing the excessive production of galactose-deficient IgA1 and subsequent heightened immune responses (33, 34). Of note, the new onset of haematuria and proteinuria was found simultaneously with a flare of DM in this patient. Therefore, the development of IgAN may also be attributed to a systemic active immune response in the patient. Additional studies are required to determine the pathogenetic mechanisms of IgAN underlying anti-TIF1-γ antibody-positive DM. On the other hand, the prevalence of IgAN varies largely according to geography and ethnicity, and IgAN is more common among Asians than in Caucasians (35). Importantly, IgAN has been reported to account for 54.3% of primary glomerular diseases in China (36). In this respect, this case study might simply reflect the higher prevalence of IgAN in China. The possibility of a coincidence finding between IgAN and anti-TIF1-γ antibody-positive DM in this patient cannot be completely ruled out. Additionally, although time intervals between the occurrence of SAMs and IgAN reported previously can be as long as 18 years (11), it might be, to some extent, more likely to be coincidental rather than causative relationship between these two diseases due to the relatively long time interval (six years) in this patient.

There are currently no guidelines for the treatment of secondary IgAN. According to the different clinical conditions of the patients, the standard treatment for primary IgAN is the use of angiotensin-converting enzyme inhibitors and steroids or even immunosuppressants. However, recent studies have shown that adding immunosuppressive therapy to the intensive supportive treatment of patients with high-risk IgAN does not significantly improve the prognosis, because the estimated glomerular filtration rate does not change and more adverse reactions have been observed (37). Accordingly, a single angiotensin receptor blocker was added to treat the IgAN in our patient due to having no crescent formation found in the kidney biopsy and a normal renal function. Compared with patients with primary IgAN, patients with secondary IgAN were older, had more complications and had higher levels of haematuria but lower levels of proteinuria. The pathological changes and clinical outcomes of secondary IgAN were similar to those in primary IgAN (38). The conclusion of this retrospective study may not be robust enough and should be interpreted with caution, due to the fact that only 16 patients with systemic autoimmune diseases were included. Intriguingly, several recent studies have demonstrated that secondary IgAN, such as spondyloarthritis-associated and IBD-associated IgAN, is related with a poor renal outcome, with an elevated risk of progression to end-stage kidney disease (17, 39). Compared with those without renal involvement, an increased risk of cardiovascular diseases was observed in RA patients with renal abnormalities (40). Taken together, it could be reasonable to postulate that IgAN secondary to systemic autoimmune diseases may have a worse prognosis than primary IgAN, probably due to the underlying systemic diseases. Therefore, careful monitoring of urinalysis and renal function should be performed routinely in patients with systemic autoimmune diseases, such as DM, for the purpose of early diagnosis of IgAN and avoiding nephrotoxic immunosuppressants. However, more studies are needed to investigate the prognosis of patients with DM and concurrent IgAN.

The main limitation of the current study is that this is a case report. Although several cases of IgAN developing in SAMs have been previously described, to the best of our knowledge, this is the first case study depicting an anti-TIF1γ antibody-positive DM patient complicated with IgAN, which indicates that IgAN may be secondary to DM with positive anti-TIF1γ antibodies. Further studies are needed to explore the relationship between SAMs and IgAN, as well as the underlying mechanisms.

## Conclusion

We describe the first case of a patient with DM with positive anti-TIF-1γ antibodies who was diagnosed with IgAN during standard immunosuppressive therapy, which adds to the small body of existing evidence on the possible association of SAMs and IgAN. Urinalysis and renal function tests should be performed routinely in patients with SAMs, even in those with anti-TIF1γ-positive DM, for an early diagnosis and management of IgAN. Further studies are needed to explore the relationship between SAMs and IgAN.

## Data Availability Statement

The original contributions presented in the study are included in the article/**Supplementary Material**. Further inquiries can be directed to the corresponding author.

## Ethics Statement

The studies involving human participants were reviewed and approved by Medical Ethics Committee of Shenzhen People’s Hospital. The patients/participants provided their written informed consent to participate in this study. Written informed consent was obtained from the individual(s) for the publication of any potentially identifiable images or data included in this article.

## Author Contributions

SZ and Y-LC summarized the case, reviewed the literature, and drafted the manuscript. C-LL gathered the clinical data. J-YX and B-DS helped draft the manuscript. D-ZL reviewed and summarized the case. All authors have read and approved the submitted version of the manuscript.

## Funding

This work was supported by Shenzhen Science and Technology Innovation Program (grant number JCYJ20190807144418845); National Natural Science Foundation of China (grant number 81971464); and National Key Research and Development Program of China (grant number 2019YFC0840603).

## Conflict of Interest

The authors declare that the research was conducted in the absence of any commercial or financial relationships that could be construed as a potential conflict of interest.

## Publisher’s Note

All claims expressed in this article are solely those of the authors and do not necessarily represent those of their affiliated organizations, or those of the publisher, the editors and the reviewers. Any product that may be evaluated in this article, or claim that may be made by its manufacturer, is not guaranteed or endorsed by the publisher.

## Supplementary Material

Supplementary Figure 1 Clinical presentation of the patient. **(A)** Heliotrope rash over the face and neck. **(C)** Gottron’s papules over the extensor surfaces of the dorsal interphalangeal and metacarpophalangeal joints. **(B, D)** Three months after treatment.
