# Supplementary figures and images for: Case Report: IgA Nephropathy in a Patient With Anti-Transcription Intermediary Factor-1γ Antibody-Positive Dermatomyositis
Source: Front Immunol. 2022 Feb 3;13:757802. doi: 10.3389/fimmu.2022.757802 (PMC8852326; doi:10.3389/fimmu.2022.757802)

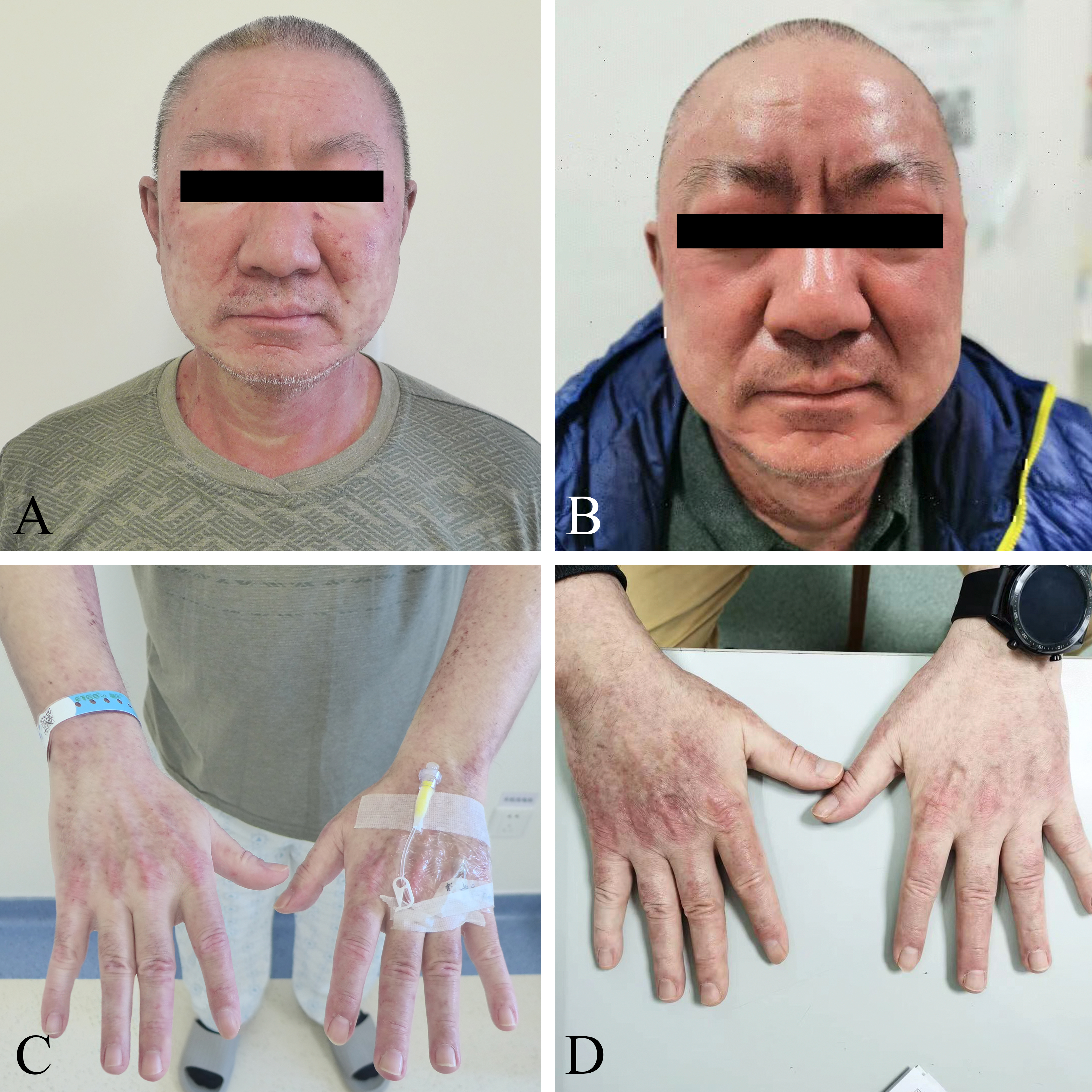

Supplement: Supplementary Figure 1 — Clinical presentation of the patient. (A) Heliotrope rash over the face and neck. (C) Gottron’s papules over the extensor surfaces of the dorsal interphalangeal and metacarpophalangeal joints. (B, D) Three months after treatment. [file Image_1.jpeg]
