# Supplementary material for: Case Report: IgA Nephropathy in a Patient With Anti-Transcription Intermediary Factor-1γ Antibody-Positive Dermatomyositis
Source: Front Immunol. 2022 Feb 3;13:757802. doi: 10.3389/fimmu.2022.757802 (PMC8852326; doi:10.3389/fimmu.2022.757802)
Supplement: Supplementary file 2 [file Table_1.docx]

Supplementary Material

**Supplementary Table 1   Laboratory findings on admission**

| **Parameter** | **Value** | **Reference range** |
| --- | --- | --- |
| Leukocytes (10^9^/L) | 3.69 | 4-10 |
| Haemoglobin (g/L) | 142 | 110-150 |
| Platelets (10^9^/L) | 214 | 100-300 |
| Glucose (mmol/L) | 5.52 | 3.9-6.1 |
| Cholesterol (mmol/L) | 5.40 | 3.4-6.5 |
| HDL (mmol/L) | 0.94 | 0.9-1.91 |
| LDL (mmol/L) | 3.39 | 2.08-4.14 |
| AST (U/L) | 59 | 0-45 |
| ALT (U/L) | 85 | 0-40 |
| CK (U/L) | 171 | 25-192 |
| LDH (U/L) | 281 | 110-240 |
| Urea nitrogen (mmol/L) | 3.99 | 2.5-7.5 |
| Creatinine (µmol/L) | 58 | 44-133 |
| IgG (g/L) | 15.68 | 8-18 |
| IgA (g/L) | 6.25 | 0.7-4.5 |
| IgM (g/L) | 0.58 | 0.4-2.5 |
| Ferritin level (ng/mL) | 708.7 | 11.0-306.8 |
| 24-h urine protein (g/24 h) | 0.94 | 0.028-0.141 |
| ESR (mm/h) | 30 | 0-20 |
| CRP (mg/L) | 3.46 | <5 |
| C3 (g/L) | 1.06 | 0.80-1.81 |
| C4 (g/L) | 0.23 | 0.15-0.57 |
| Coombs’ test | Negative | Negative |
| ANA | Negative | Negative |
| cANCA | Negative | Negative |
| pANCA | Negative | Negative |
| anti-PR3 | Negative | Negative |
| anti-MPO | Negative | Negative |
| anti-GBM | Negative | Negative |
| Anti-dsDNA | Negative | Negative |
| Anti-SS-A | Negative | Negative |
| Anti-SS-B | Negative | Negative |

HDL, high-density lipoprotein; LDL, low density lipoprotein; ALT, alanine aminotransferase; AST, aspartate transaminase; CK, creatine phosphokinase; LDH, lactic dehydrogenase; Ig: immunoglobulin; ESR, erythrocyte sedimentation rate; CRP, C-reactive protein; C3, complement C3; C4, complement C4; ANA, anti-nuclear antibodies; cANCA, cytoplasmic-staining anti-neutrophil cytoplasm antibody; pANCA, perinuclear-staining anti-neutrophil cytoplasm antibody; Anti-MPO, anti-myeloperoxidase antibody; Anti-PR3, anti-proteinase 3 antibody; anti-GBM, anti-glomerular basement membrane antibody; Anti-dsDNA, anti-double-stranded DNA antibody; Anti-SS-A, anti-Sjögren’s syndrome antigen A antibody; Anti-SS-B, anti-Sjögren’s syndrome antigen B antibody.
